# Ameliorative Effects and Possible Molecular Mechanism of Action of Black Ginseng (*Panax ginseng*) on Acetaminophen-Mediated Liver Injury

**DOI:** 10.3390/molecules22040664

**Published:** 2017-04-21

**Authors:** Jun-Nan Hu, Zhi Liu, Zi Wang, Xin-Dian Li, Lian-Xue Zhang, Wei Li, Ying-Ping Wang

**Affiliations:** College of Chinese Medicinal Materials, Jilin Agricultural University, Changchun 130118, China; junnanhu005@126.com (J.-N.H.); lzhiiu@126.com (Z.L.); wangzi8020@126.com (Z.W.); xdli2005@126.com (X.-D.L.); zlxbooksea@163.com (L.-X.Z.)

**Keywords:** black ginseng, APAP, liver injury, apoptosis, oxidative stress

## Abstract

*Background*: Frequent overdosing of acetaminophen (APAP) has become the major cause of acute liver injury (ALI). The present study aimed to evaluate the potential hepatoprotective effects of black ginseng (BG) on APAP-induced mice liver injuries and the underlying mechanisms of action were further investigated for the first time. *Methods*: Mice were treated with BG (300, 600 mg/kg) by oral gavage once a day for seven days. On the 7th day, all mice were treated with 250 mg/kg APAP which caused severe liver injury after 24 h and hepatotoxicity was assessed. *Results*: Our results showed that pretreatment with BG significantly decreased the levels of serum alanine aminotransferase (ALT) and aspartate transaminase (AST) compared with the APAP group. Meanwhile, hepatic antioxidant including glutathione (GSH) was elevated compared with the APAP group. In contrast, a significant decrease of the levels of the lipid peroxidation product malondialdehyde (MDA) was observed in the BG-treated groups compared with the APAP group. These effects were associated with significant increases of cytochrome P450 E1 (CYP2E1) and 4-hydroxynonenal (4-HNE) levels in liver tissues. Moreover, BG supplementation suppressed activation of apoptotic pathways through increasing Bcl-2 and decreasing Bax protein expression levels according to western blotting analysis. Histopathological examination revealed that BG pretreatment significantly inhibited APAP-induced necrosis and inflammatory infiltration in liver tissues. Biological indicators of nitrative stress like 3-nitrotyrosine (3-NT) were also inhibited after pretreatment with BG, compared with the APAP group. *Conclusions*: The results clearly suggest that the underlying molecular mechanisms of action of BG-mediated alleviation of APAP-induced hepatotoxicity may involve its anti-oxidant, anti-apoptotic, anti-inflammatory and anti-nitrative effects.

## 1. Introduction

The liver is an important metabolic organ responsible, for the clearance of numerous toxins, drugs and pathogens, but it can also be injured by these harmful substances [1]. Nowadays, acute hepatic failure has become one of the major diseases affecting human health around the world. It can be attributed to various factors such as hepatitis virus infection, and induction by drugs and toxins [2]. Acetaminophen (APAP), a widely used analgesic and antipyretic drug, is safe and effective when taken at therapeutic doses [3]. However, acetaminophen overdosing can cause hepatotoxicity and nephrotoxicity [4]. Widespread use of APAP in hundreds of prescription and over-the-counter drugs has increased the prevalence of APAP hepatotoxicity [5,6].

Generally, APAP is metabolized primarily in the liver via glucuronidation and sulfation [7]. APAP is also oxidized by cytochrome P450 enzymes to its chemically reactive metabolite which can cause liver and kidney damages [8]. *N*-Acetyl-*p*-benzoquinone imine (NAPQI) is known as the putative reactive metabolite of APAP. When APAP is at a high level, the accumulation of NAPQI, will result in increased utilization of GSH and depletion of GSH stores [9]. Once intracellular GSH stores are depleted, excess NAPQI that is not conjugated with GSH may react with cellular proteins, including mitochondrial proteins [10]. This situation leads to the formation of reactive oxygen and nitrogen species, and initiates lipid peroxidation that eventually results in liver cell destruction, necrosis, or apoptosis [11,12]. Although in clinical and experimental studies, many compounds have been investigated for their ability to protect against APAP-induced hepatotoxicity [13,14], the need for new treatment options remains high.

The roots of *Panax ginseng* C. A Meyer (ginseng), one of the best known Traditional Chinese Medicines, have been reported to display adaptogenic effects in the endocrine, immune, cardiovascular, and central nervous systems [15,16]. Generally, the ginsenosides, which possess many pharmacological effects including anti-diabetes, anti-tumor, and anti-inflammation activity are considered the major active constituents in ginseng [17]. Previous studies have demonstrated that the pharmacological and biological activities of steamed-processed ginseng (red ginseng and black ginseng) are greater than those of non-steamed (white ginseng) [18]. The steaming process could lead to extensive conversion of the ginsenosides in non-steamed ginseng into new less polar degradation products such as ginsenoside Rg3, Rg5, Rk1, Rz1, F4, and Rg6 [19,20]. Black ginseng (BG), generated from ginseng through steaming and drying, is confirmed to exert many pharmacological effects, such as anti-cancer, anti-inflammatory, and anti-antioxidant actions [21]. Though fermented ginseng (which also contains many less polar ginsenosides) was reported to protect against APAP-induced hepatotoxicity in a rat model [22], the protective effect of BG on APAP-induced liver injury had not been reported so far. Therefore, it was of great interest to investigate the effect of BG on APAP-induced liver injuries.

On the basis of the above facts and taking into account that APAP overdose frequently results in life-threatening hepatotoxicity, we decide to explore the potential ameliorative effect of BG in a mouse model of APAP-induced liver hepatotoxicity. Importantly, to the best of our knowledge, the potential mechanisms underlying such hepatoprotective effects of BG are revealed in this work for the first time.

## 2. Results

### 2.1. HPLC Analysis of Ginsenosides in Black Ginseng

Ginsenosides are the major active constituents of ginseng (*Panax ginseng*) responsible for its numerous pharmacological functions [23]. As we know, the rare ginsenosides, including Rg3, Rk1, and Rg5 are considered to possess stronger activities than the original ginsenosides. Black ginseng, obtained from sun-dried ginseng via repeated (nine times) steaming at 95 °C for 3 h, and drying at 60 °C for 12–24 h [24], contains more rare ginsenosides such as 20(s)-Rg3, 20(r)-Rg3, Rk1 and Rg5.

**Figure 1:**
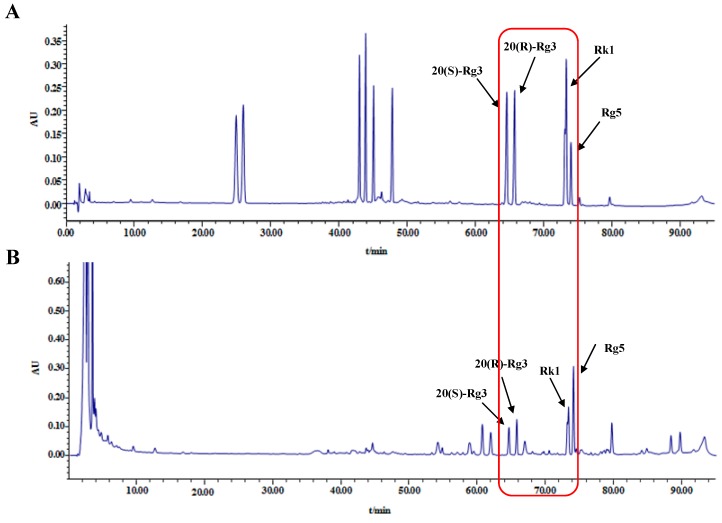
HPLC chromatogram of ginsenosides in mixed standard compounds (**A**) and Black ginseng (**B**).

Since the BG was derived from the heat-processed ginseng and exhibits the strong antioxidant effects [21], a simple HPLC method was used to identify the ginsenosides content in BG. As shown in Figure 1, the total contents of 20(s)-Rg3, 20(r)-Rg3, Rk1, and Rg5 were 69.33 mg/g.

### 2.2. Effect of Black Ginseng on Body Weight and Organ Indices

Organ indices of the liver and spleen were evaluated in mice. As shown in the Table 1, APAP-treated mice gained less weight than the normal group. Liver and spleen indices were significantly increased in mice that were exposed to APAP (*p* < 0.01, *p* < 0.05). However, in the two treatment groups, these indices were significantly reduced (*p* < 0.01, *p* < 0.05) compared to the APAP group. In addition, there was no difference in the kidney indices among all groups.

### 2.3. Effect of Black Ginseng on Serum Biochemical Markers

**Figure 2:**
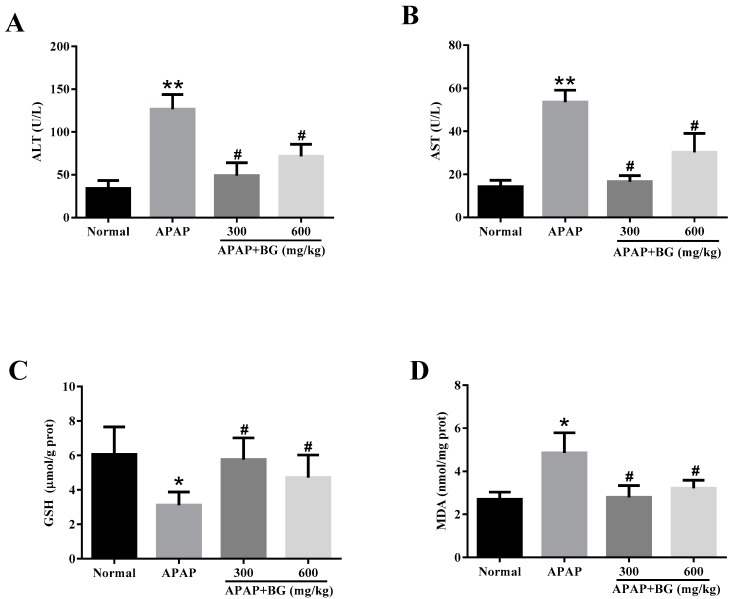
Effects of BG on levels of ALT (**A**) and AST (**B**) in serums, and GSH (**C**) and MDA (**D**) in liver tissues of mice. Values are expressed as the mean ± S.D., *n* = 8; ** *p* < 0.01, * *p* < 0.05 vs. normal group; ^#^ *p* < 0.05 vs. APAP group.

As showed in Figure 2A,B, the serum levels of ALT and AST were evaluated to determine the degree of liver damage. Compared to the normal group, the serum levels of ALT and AST were significantly elevated after APAP challenge (*p* < 0.01), indicating hepatocellular damage and confirming the mouse model of APAP-induced liver injury had been established successfully. However, the serum levels of ALT and AST in APAP + BG (300 mg/kg or 600 mg/kg) groups decreased significantly (*p* < 0.05), and the low-dose group (300 mg/kg) showed a better result.

### 2.4. Effect of Black Ginseng on Attenuates APAP-Induced Oxidative Stress 

The levels of GSH and MDA caused by the administration of APAP to mice are summarized in Figure 2. As shown in Figure 2C, compared to the normal group, the hepatic level of GSH in the APAP group were decreased observably (*p* < 0.05), and pretreatment with BG for seven days completely reserved the decrease (*p* < 0.05). As shown in Figure 2D, compared with that in the normal group, the level of MDA in the APAP group was significantly elevated (*p* < 0.05), indicating oxidative injury after APAP exposure. However, the administration of BG with 300 mg/kg and 600 mg/kg could protect against the APAP-induced elevation of MDA level (*p* < 0.05). These data demonstrated clearly that BG alleviated liver oxidative stress injuries.

**Figure 3:**
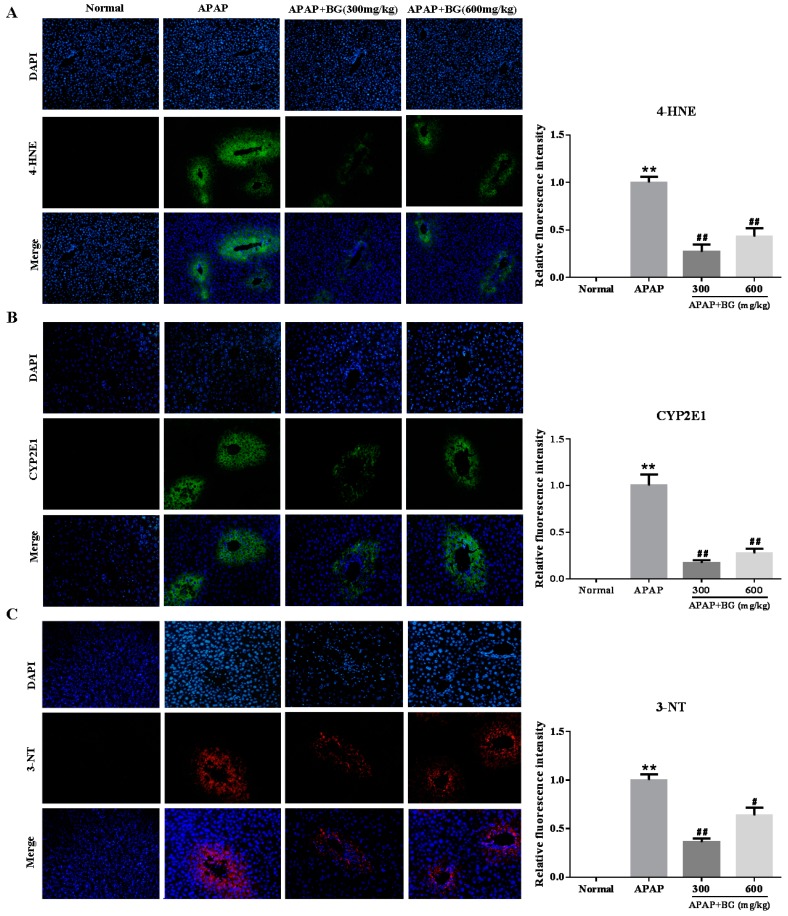
Effects of BG on expression of 4-hydroxynonenal (4-HNE) (**A**), cytochrome P450 E1 (CYP2E1) (**B**) and 3-nitrotyrosine (3-NT) (**C**) in liver tissues, and the fluorescence intensities were quantified. The expression levels of 4-HNE, CYP2E1 (green) and 3-NT (red) in tissue section isolated from different groups were assessed by immunofluorescence. Representative immunofluorescence images were taken at 200×. 4,6-Diamidino-2-phenylindole (DAPI) (blue) was used as a nuclear counterstain. All data were expressed as mean ± S.D, *n* = 8. ** *p* < 0.01 vs. normal group; ^##^ *p* < 0.01, ^#^ *p* < 0.05, vs. APAP group.

To further confirm whether or not oxidative stress is involved in the development of APAP-induced hepatotoxicity in vivo, lipid peroxidation was confirmed again using 4-HNE staining. After APAP exposure for 24 h, strong 4-HNE fluorescence intensities were detected in the cytoplasm of the liver of mice at 24 h after APAP exposure. However, BG pretreatment for seven days could decrease these fluorescence intensities significantly, especially in the low-dose group (300 mg/kg) (Figure 3A). Interestingly, the sites of lipid peroxidation showed high correlations with liver necrotic regions.

Since CYP-mediated bioactivation is considered to play an important role in APAP-induced hepatotoxicity, the protein expression of CYP2E1 in liver tissues was checked at 24 h after APAP challenge. As expected, the expression of the CYP2E1 metabolizing enzyme was significantly increased after APAP challenge for 24 h. However, treatment with BG could decrease the expression of CYP2E1 (Figure 3B). These results also suggest that pretreatment of BG attenuates to some extent APAP-induced oxidative stress injury.

### 2.5. Effect of Black Ginseng on Expression of 3-NT in Liver tissues

Nitration of tyrosine (i.e., formation of NT), shown to be an excellent biomarker of peroxynitrite formation, was found in the centrilobular cells of APAP-treated mice liver [25]. In our study, livers from APAP-treated mice were examined by immunofluorescence analyses for 3-nitrotyrosine-protein adducts. As shown in Figure 3C, APAP-treated mice livers stained positive for 3-NT in all the observed centrilobular areas. In contrast, the group of mice treated with BG presented less 3-NT staining. The results implied that pretreatment with BG might alleviate APAP-induced liver injury by suppressing nitrative stress.

### 2.6. Effect of Black Ginseng on Liver Histopathology

**Figure 4:**
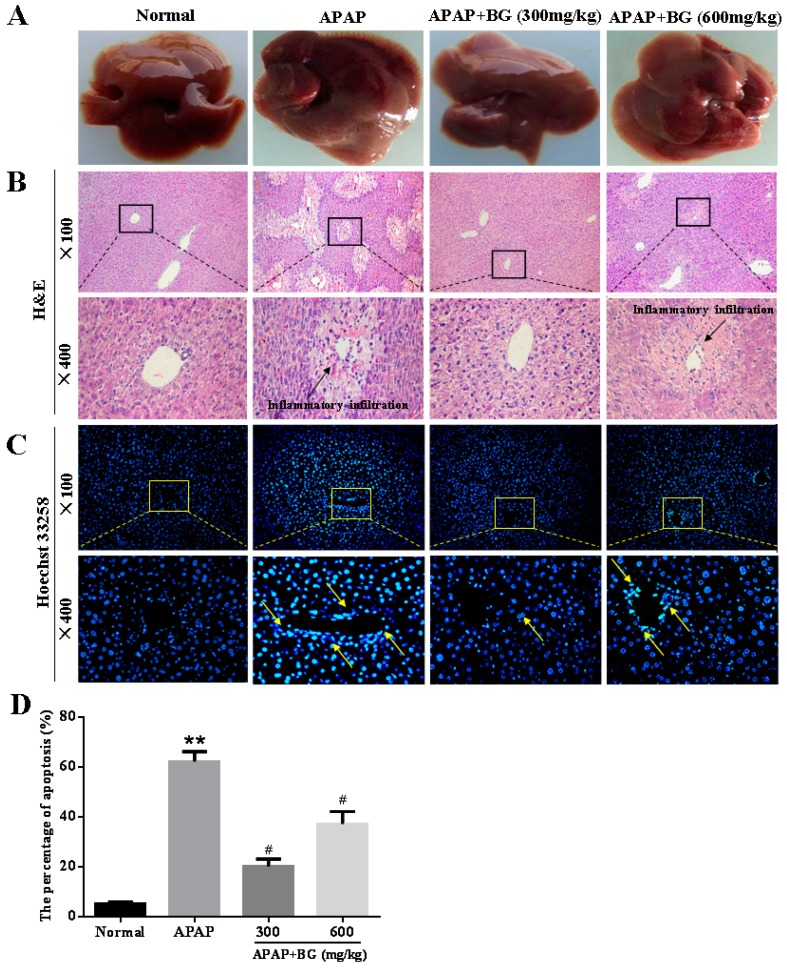
Histological examination of morphological changes in liver tissues. Pathological change of livers (**A**); and liver tissues stained with H&E (**B**); and Hoechst 33258 (**C**); and the percentage of apoptosis (**D**). Arrows show necrotic and injured cells. All data were expressed as mean ± S.D, *n* = 8. ** *p* < 0.01 vs. normal group; ^#^ *p* < 0.05, vs. APAP group.

The livers of the four groups were observed (Figure 4A). The livers of APAP-treated mice were more haemorrhagic than those of the normal group, and pretreatment with BG ameliorated the injuries induced by APAP. Liver sections from all four groups were examined by H&E staining. As shown in Figure 4B and Table 2, the hepatic sections in the normal group had clear hepatic lobule and regular hepatic cord structures with central veins, and the cell nucleus was normal. However, in the APAP group, typical pathological characteristics including necrosis and inflammatory infiltration confirmed the successful establishment of the liver injury model. Pretreatment with BG before APAP exposure noticeably attenuated the number of apoptotic cells and inflammation level. Interestingly, the pathological features of the APAP + BG (300 mg/kg) group were almost similar to those of the normal group. From these results, we speculate that BG pretreatment might alleviate APAP-induced liver injury.

### 2.7. Effect of Black Ginseng on Hepatocyte Apoptosis in Mice

Hoechst 33258 staining was performed to determine whether BG treatment protects against cell apoptosis in APAP-induced liver damage. As shown in Figure 4C,D, the hepatocytes of the normal group were morphologically intact, neatly arranged with a clear outline, and the chromatin was stained evenly and slightly. However, the distribution of a large area and high density of blue apoptopic liver cells and small chunks of nucleus were observed clearly in APAP group, indicating severe apoptosis after APAP challenge. Importantly, after a seven-day continuous treatment with BG (300 mg/kg and 600 mg/kg), the apoptosis was obviously alleviated and the liver damage was effectively improved.

**Figure 5:**
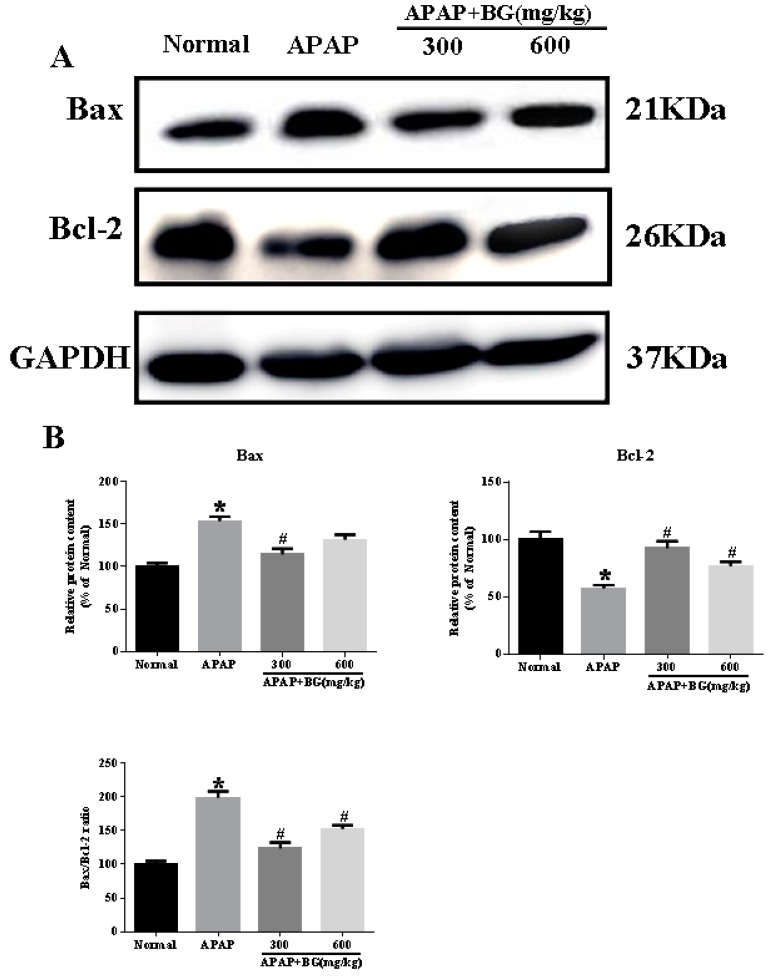
Effects of BG on the protein expression of Bax; and Bcl-2 (**A**); and results are quantified from their band intensities (**B**). The protein expression was examined by western blotting analysis in liver tissues from normal, APAP, APAP + BG (300 mg/kg), and APAP + BG (600 mg/kg) group animals. All data were expressed as mean ± S.D., *n* = 8. * *p* < 0.05 vs. normal group; ^#^ *p* < 0.05 vs. APAP group.

In the present investigation, western blotting analysis was used to further determine the impacts of BG on the pro-apoptotic factor Bax and anti-apoptotic factor Bcl-2 in all experimental groups in order to test the extent of apoptosis in liver tissues. As depicted in Figure 5A,B, pretreatment with 300 and 600 mg/kg BG decreased the protein expression of Bax and ratio of Bax/Bcl-2, while Bcl-2 protein expression increased. The results above showed that BG pretreatment might alleviate APAP-induced hepatocyte apoptosis.

### 2.8. Effect of Black Ginseng on Expression of iNOS and COX-2 in Liver Tissues

**Figure 6:**
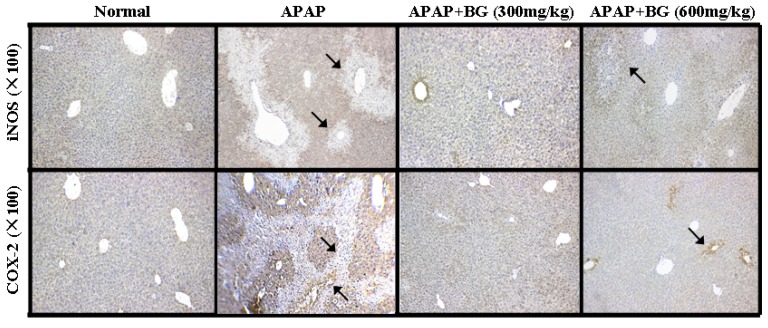
Effects of BG on expression of COX-2 and iNOS in liver tissues. Arrows show necrotic and inflammatory cells.

In order to measure the extent of inflammation in liver tissues, we examined the impact of BG on the inflammatory cytokines of iNOS and COX-2 using immunohistochemical analysis. As shown in Figure 6, compared to the normal group, iNOS and COX-2 positive expression sowed a large increasing area and was distributed around the central veins with nuclei form. Interestingly, treatment with different doses of BG for continuous seven days could reduce the positive expression. It can be confirmed that BG was playing a significant role in relieving APAP liver damage, which is a complex phenomenon involving multiple cellular and molecular interactions, and inflammatory processes, which must be rigorously controlled to avoid different pathologies and disorders.

## 3. Discussion

Acetaminophen (APAP) is the most widely used over-the-counter analgesic and antipyretic medication in the world, with few side effects when taken in therapeutic doses. However, long-term or overdose usage of APAP can result in inflammation and necrosis of hepatocytes or even acute liver failure [26]. Generally, APAP administration has been recognized as one of the most widely used in vivo animal models to induce liver injury to evaluate the hepatoprotective effects of natural medicines [27].

Ginseng, the roots of *Panax ginseng*, C.A. Meyer., is a Traditional Chinese Medicine widely used in China and Korea due to its good pharmacological activity. It has been demonstrated that steamed ginseng (e.g., red ginseng and black ginseng) exhibit more remarkable pharmacological activities and therapeutic efficacy than non-steamed ginseng (e.g., white ginseng) [28]. The differences in their biological effects can be attributed to significant changes in the ginsenosides profile during steaming [29]. Black ginseng (BG), produced from ginseng through steaming nine times and drying, respectively, shows various pharmacological effects, such as anti-cancer, anti-inflammatory, anti-stress and antioxidant properties [21,30]. Although it was reported that fermented ginseng containing more rare ginsenosides and could alleviate APAP-induced liver injury in a rat model [22], the protective effect and the possible molecular mechanisms of action of BG on APAP-induced liver injury were still not clear. Therefore, the aim of the present study was to investigate the hepatoprotective effect and underlying mechanisms of BG on APAP-induced liver injury in mice.

Serum ALT and AST are the most sensitive biomarker enzymes used in the evaluation of acute liver injury. In the present study, we have observed significant increases in serum ALT and AST activities in APAP-induced mice compared with the normal group. The results in the present study showed that BG inhibited the serum levels of ALT and AST, indicating a significant protective effect of BG against APAP-induced liver damage, but this effect was not dose-dependent, which may be ascribed to the toxicity of the saponin ingredients found in high doses of BG that has been reported in former studies [31]. Additionally, during the processing of BG, Maillard reactions can occur and produce some toxic byproducts [32], which might account for the lower efficacy of BG at a high dose.

Oxidative stress, acts as an important pathogenic factor in APAP-induced hepatotoxicity, as reported in numerous animal models [33]. APAP is metabolically activated by cytochrome P450 to generate the toxic metabolite NAPQI, which is immediately conjugated with GSH [34]. Therefore, a high overdose of APAP may cause dramatic GSH depletion in the liver, affecting liver functions and leading to massive hepatocyte necrosis, liver failure or death [35]. The MDA level is widely used as a marker of free radical mediated lipid peroxidation (LPO) injury [36]. Previous reports confirmed that an elevated MDA level in liver tissues indicated that enhanced LPO can lead to tissue damage and the breakdown of the natural antioxidant defense mechanisms [37]. In addition, there is also evidence of APAP-induced liver injury via an oxidative stress mechanism caused by increased MDA and reduced GSH levels [38]. Interestingly, the results clearly exhibited that the MDA level increased significantly and the GSH level decreased significantly after APAP challenge and pre-treatment with two doses of BG effectively alleviated these alterations and reversed the oxidative stress levels. Therefore, the increase of GSH, and decreased level of MDA in liver tissues treated with BG suggested a host-detoxification process of BG in APAP-induced liver injury. Drug-metabolizing enzyme CYP2E1, serving as a site for the generation of reactive oxygen species (ROS), plays an important role in APAP-induced hepatotoxicity [39]. In the present investigation, overexpression of CYP2E1 in liver tissues treated with APAP could be reversed after treatment with BG. Consistent with the MDA result, although 4-HNE immunofluorescence staining exhibited high fluorescence intensity in liver tissues after APAP exposure, this process was significantly blocked by pretreatment with BG.

Nitration of tyrosine (i.e., formation of NT) has been shown to be an excellent biomarker of peroxynitrite formation, and it is generally accepted that NT occurs in the centrilobular cells of the liver in overdose APAP-challenge mice. Peroxynitrite formation is formed by a rapid reaction between nitricoxide and superoxide, and peroxynitrile production was increased under APAP-toxicity conditions [40]. Peroxynitrite initiates necrotic cell death in APAP hepatotoxicity associated with DNA damage through mitochondrial dysfunction [41]. Our study clearly showed overexpression of 3-NT in liver tissues of APAP-treated mice, which was in line with the above findings. However, BG pretreatment at two doses reversed the APAP-mediated increase in 3-NT expression.

Generally, apoptosis is characterized by a fundamental cellular activity to maintain the physiological balance of the organism [42]. Accumulating evidence has indicated that APAP-induced acute liver injury was associated with cell apoptosis. Apoptosis is an especially important form of cell death, and more and more reports have demonstrated apoptosis of hepatocytes after APAP exposure [43]. Two important members of the Bcl-2 family involved in apoptosis are the pro-apoptotic protein Bax and anti-apoptotic protein Bcl-2 [44]. The findings of a western blotting analysis of liver tissues obviously indicated that the protein expression of Bax and Bax/Bcl-2 ratio were significantly suppressed while the protein expression of Bcl-2 was relatively increased, clearly indicating that BG exhibits anti-apoptotic properties in the context of APAP hepatotoxicity. In addition, Hoechst 33258 staining was employed to observe the apoptotic liver cells in the APAP-induced liver injury model. The results clearly showed a large area and high density of blue apoptosis staining, indicating the apoptosis of liver cells. However, the apoptosis in the BG groups was obviously alleviated and the liver damage was effectively improved, which confirmed that BG treatment could protect against APAP-induced liver cell apoptosis.

Recent evidence has indicated that inflammation plays an important role in the pathogenesis of APAP-induced liver injury. Therefore, overproduction of inflammatory cytokines could be considered a precursor to liver diseases, and the down-regulation of these inflammatory molecules is considered a beneficial effect [14]. COX-2 and iNOS are enzymes involved in the development of inflammatory process development [45]. In addition, the induction of COX-2, an inducible form of COX, can occur during tissue damage or inflammation in response to cytokines. It can be speculated that COX-2 plays a pivotal role in APAP-induced acute liver injury. Our immunohistochemical results revealed that the expression of iNOS and COX-2 was significantly increased in the APAP-injured mice. However, treatment with BG could effectively inhibit the overexpression of iNOS and COX-2 in liver tissues.

## 4. Materials and Methods

### 4.1. Plant Materials

The black ginseng (BG) was generated from the roots of *P. ginseng* and identified by Professor Wei Li (College of Chinese Medicinal Material, Jilin Agricultural University, Changchun, China). After cutting the black ginseng into slices, the cut pieces were ground to obtain a relatively homogenous drug powder and then sieved through a 40-mesh screen. The powder was dried at 60 °C until its weight remained constant and was well blended before use.

### 4.2. Chemicals and Reagents

Acetaminophen (>99.0%, batch No. A105808-25 g) was purchased from Aladdin Industrial Corporation (Shanghai, China). The kits for detection of alanine aminotransferase (ALT), aspartate transaminase (AST), glutathione (GSH), and malondialdehyde (MDA) were purchased from Nanjing Jiancheng Bioengineering Research Institute (Nanjing, China). Rabbit monoclonal anti-mouse iNOS, COX-2, Bax, Bcl-2, cytochrome P450 E1 (CPY2E1), 4-hydroxynonenal (4-HNE) and 3-nitrotyrosine (3-NT) were purchased from Cell Signaling Technology (Danvers, MA, USA). Hoechst 33258 staining kit was purchased from Beyotime Institute of Biotechnology (Shanghai, China). DyLight 488-SABC and SABC-Cy3 immunofluorescence Staining kit was purchased from BOSTER Biological Technology (Wuhan, China). All other reagents and chemicals were of analytical grade and purchased from Beijing Chemical Factory (Beijing, China).

### 4.3. Identification and Analysis of Ginsenosides in Black Ginseng

Sample extraction was performed as previously described with some modifications [46]. About 1.0 g of powdered BG was extracted two times with 100% methanol by ultrasonic-assisted extraction for 30 min. The combined extracts were concentrated with an evaporator, then diluted with methanol to 5.0 mL in a volumetric flask, the supernatant was then filtered through a 0.45 μm nylon membrane and injected into the HPLC for analysis.

Samples were analyzed on an Agilent 1200 HPLC system (Agilent Technologies, Santa Clara, CA, USA) equipped with a UV detector. Liquid chromatographic separations were achieved using a Hypersil ODS2 column (4.6 cm× 25 cm, 5 μm). The column temperature was set at 30 °C and the detection wavelength was set at 203 nm. The mobile phase consisted of a mixture of acetonitrile (A) and water (B) with a flow rate of 1.0 mL/min. The gradient elution was programmed as follows: 0–20 min, 18.5–20.5% A; 20–30 min, 20.5–25.5% A; 30–40 min, 25.5–35% A; 40–60 min, 35–45% A; 60–70 min, 45–60% A; 70–80 min, 60–70% A; 80–90 min, 70–80% A. The 20 μL sample solutions were directly injected into the chromatographic column manually.

### 4.4. Animals

Thirty-two male ICR mice, weighing 22–25 g, were provided by Experimental Animal Holding of Yisi Experimental Animals with Certificate of Quality No. of SCXK (JI) 2016-0003 (Changchun, China). The animals were housed in plastic cages under standard laboratory conditions (12 h light/dark cycle, relative humidity 60% ± 5%, and 25 ± 2 °C) and adaptability raise at least one week before commencement of the experiment. All animals handing procedures were performed in strict accordance with the Guide for the Care and Use of Laboratory Animals (Ministry of Science and Technology of China, 2006). All experimental procedures were approved by the Ethical Committee for Laboratory Animals of Jilin Agricultural University (Permit Number: 16-008)

### 4.5. Experimental Groups and Treatment

The animals were randomly divided into four groups (eight animals per group) as follows: normal, APAP (250 mg/kg), APAP + BG (300 mg/kg) and APAP + BG (600 mg/kg). The treatment groups were administered BG by gastric intubation for seven consecutive days at doses of 300 mg/kg and 600 mg/kg body weight per day, and the normal and APAP groups were treated with 0.9% saline in the same way. After final administration, animals in APAP and BG-treatment groups received a single intraperitoneal (i.p.) injection of APAP (250 mg/kg) to induce acute liver injury in mice. All the mice were fasted for at least 12 h before intraperitoneal injected with APAP and dissected, they were allowed free access to water. Then, all the mice were killed by means of cervical vertebra dislocation, and their blood was collected. The serum was separated by centrifugation (3500 rpm, 10 min, and 4 °C) and stored at −20 °C for the determination of measuring aminotransferases. All the mice were sacrificed and the segregated livers were washed twice with saline, blotted dry on a filter paper, weighed. At the same time, shapes, colors and sizes of the livers were observed. Livers were dissected quickly, a small piece of tissue was cut off from the same part of the left lobe of the liver in each mouse and fixed in 10% buffered formalin solution (*m*/*v*) for histopathological analysis. The remaining liver tissues were stored at −80 °C for hepatic homogenate preparation.

### 4.6. Assay for Hepatic Function Biochemical Markers

To assess hepatic function, serum was used for the spectrophotometric determination of ALT and AST using commercially available detection kits according to the manufacturers’ instructions. In brief, the samples were transferred into a new 96-well plate containing substrates or buffer solution. After incubation at 37 °C, the plate was incubated for an additional time after adding color developing agent and the absorbance at 510 nm was measured. The final data are represented as U/L.

### 4.7. Assay for GSH and MDA in Liver

The liver tissues were homogenated in 50 mM phosphate buffer (pH 7.4). The resulting suspension was then centrifuged at 3500 r for 15 min at 4 °C, and the supernatant was used for the detection of GSH and MDA. In brief, the levels of GSH and MDA in liver homogenates were measured by commercial kits according to the manufacturer’s instructions [47]. The amount of protein was measured using the Bradford assay [48].

### 4.8. Histopathological Examination

For histopathological analysis, the liver tissues (n = 8 per group) were fixed in 10% buffered formaldehyde for over 24 h, subsequently processed by routine paraffin embedding and sectioned for 5 μm thickness. After hematoxylin-eosin (H&E) staining, slides were observed for histopathological changes using a light microscope (Leica, Wetzlar, Germany). The degree of hepatocellular necrosis was analyzed by Ridit analysis [49].

### 4.9. Hoechst 33258 Staining

Hoechst 33258 staining was performed as previously described with some modifications [50]. Briefly, at the end of the experiments, the liver tissues were dissected out and fixed in 10% neutral buffered formalin solution. We randomly chose three from each group. Then, these samples were cut into 5 μm sections and stained by Hoechst 33258 (10 μg/mL). After being washed with PBS for three times, stained nuclei were visualized under UV excitation and photographed under a fluorescence microscope (Leica TCS SP8). Image-Pro plus 6.0 was used to quantify the Hoechst 33258 staining.

### 4.10. Immunohistochemistry (IHC) and Immunofluorescence Analysis

Immunohistochemical staining was performed as previously described [51]. Briefly, the 5 μm thick paraffin sections were deparaffinized and rehydrated with a series of xylene and aqueous alcohol solutions, respectively. After antigen retrieval in citrate buffer solution (0.01 M, pH 6.0) for 20 min, the slides were washed with TBS (0.01 M, pH 7.4) for three times and incubated with 1% bovine serum albumin for 1 h. The blocking serum was tapped off, and the sections were incubated in a humidified chamber at 4 °C overnight with primary antibodies including mouse polyclonal anti-iNOS (1:200) and anti-COX-2 (1:200), followed by secondary antibody for 30 min. Substrate was added to the sections for 30 min followed by DAB staining and hematoxylin counter-staining. The positive staining was determined mainly by a brownish-yellow color in the cytoplasm or nucleus of the cells. An image was taken by light microscopy (Olympus BX-60, Tokyo, Japan).

To assess the expression of CYP2E1, 4-HNE, and 3-NT in APAP-induced hepatotoxicity, immunofluorescence staining was exerted in liver tissues sections of APAP-induced groups and normal group as described for IHC analysis. In brief, the liver sections were taken microwave antigen repaired after deparaffinized and rehydrated, added serum sealing fluid for 20 min when sections reach room temperature, dilute primary mouse polyclonal anti- CYP2E1 (1:200), anti-4-HNE (1:100) and anti-3-NT (1:200) were added and keep 4 °C overnight. The sections washed with TBS (0.01 M, pH 7.4) three times, 2 min each and subsequently marked with fluorescence secondary antibody for 30 min at room temperature, then exposed by dilute DyLight 488-SABC (1:400) or CY3 (1:100) before 30 min incubation at 37 °C. The sections were washed with TBS (0.01 M, pH 7.4) for four times, 5 min every time. Nuclear staining was performed using 4, 6 diamidino-2-phenylindole (DAPI). Immunofluorescence staining was visualized using a Leica microscope (Leica TCS SP8), and immunofluorescence intensity were analyzed by Image-Pro Plus 6.0 software.

### 4.11. Western Blotting Analysis

Western blot analysis was performed as described previously [52]. Briefly, the liver tissues were lysed in RIPA buffer and protein concentrations were measured by BCA protein assay kit (Beyotime Biotechnology, Shanghai, China). Equal amounts of protein (50 µg/lane) were analyzed by 12% SDS-PAGE and transferred onto PVDF membrane, then blocked with 5% non-fat milk in Tris-buffered saline (TBS) containing 0.1% Tween-20. After incubating with primary antibodies against Bax (1:2000) and Bcl-2 (1:2000) at 4 °C overnight, the membrane was then incubated with the secondary antibodies for 1 h at room temperature. Signals were detected using ECL substrate (Pierce Chemical Co., Rockford, IL, USA). Expression in the protein levels were measured by western blotting using Bio-Lad Laboratories-Segrate lately (Bio-Rad Laboratories, Hercules, CA, USA). The bands intensities were quantified by densitometry.

### 4.12. Statistical Analysis

All the results were expressed as means ± standard deviation (S.D). The data were analyzed using two-tailed test or one-way analysis of variance (ANOVA). GraphPad Prism 6.0 (ISI^®^, Philadelphia, PA, USA) was employed to make resulting data chart. The Nonparametric Test (Ridit analysis) was used for the histological examination comparison. *p* < 0.05 or *p* < 0.01 was considered to be significant. 

## 5. Conclusions

Taken together, our findings clearly demonstrate that BG can protect against APAP-induced hepatotoxicity in mice via attenuation of oxidative stress and nitrative stress, suppression of inflammation and apoptosis. Importantly, these data strongly suggest that BG could be an effective supplementary drug for the treatment and prevention of liver injury.

## Figures and Tables

**Table 1:** Effects of BG on body weights and organ indices in mice.

Groups	Dosage (mg/kg)	Body Weights (g)		Organ Indices (mg/g, ×100)		
		Initial	Final	Liver	Spleen	Kidney
Normal	-	28.92 ± 1.12	28.95 ± 2.13	5.71 ± 0.87	0.43 ± 0.06	1.65 ± 0.18
APAP	-	28.74 ± 1.36	27.02 ± 1.26	7.30 ± 0.78 **	0.53 ± 0.09 *	1.54 ± 0.35
APAP + BG	300	28.76 ± 1.54	28.78 ± 1.62	4.95 ± 0.62 ^##^	0.44 ± 0.03 ^#^	1.47 ± 0.12
APAP + BG	600	28.81 ± 1.79	28.53 ± 1.71	5.02 ± 0.35 ^##^	0.33 ± 0.05 ^##^	1.45 ± 0.13

Values are expressed as the mean ± S.D., *n* = 8; * *p* < 0.05, ** *p* < 0.01 vs. normal group; ^#^ *p* < 0.05, ^##^ *p* < 0.01 vs. APAP group.

**Table 2:** Pathological changes in the liver and Ridit analysis.

Groups	Dosage (mg/kg)		Necrocytosis Grade					Score	Ridit Analysis
		*n*	0	1	2	3	4		
Normal	-	8	8	0	0	0	0	0	0.27
APAP	-	8	0	2	1	4	1	20	0.81 **
APAP + BG	300	8	5	2	1	0	0	4	0.41 ^##^
APAP + BG	600	8	4	1	2	1	0	8	0.51 ^##^

Note: The necrocytosis was classed on the basis of the H&E staining of liver sections. The data were analyzed by Ridit analysis. Values represent the mean ± S.D., *n* = 8. ** *p* < 0.01 vs. normal group; ^##^ *p* < 0.01 vs. APAP group. Grading standard: level 0 means have no necrocytosis, normal in the cell of liver; level 1 means liver cells containing necrocytosis of no more than 1/4; level 2 means liver cells containing necrocytosis of no more than 1/2; level 3 means liver cells containing necrocytosis of no more than 3/4; level 4 means liver tissue was almost all of necrocytosis. Level 0 calculated 0 mark; Level I calculated 1 mark; Level II calculated 2 marks; Level III calculated 3 marks; Level IV calculated 4 marks.

## Abbreviations

The following abbreviations are used in this manuscript:

BG	Black ginseng
APAP	Acetaminophen
ALT	Alanine aminotransferase
AST	Aspartate aminotransferase
GSH	Glutathione
MDA	Malondialdehyde
iNOS	Inducible nitric oxide synthase
COX-2	Cyclooxygenase-2
3-NT	3-Nitrotyrosine
4-HNE	4-hydroxynonenal
CYP2E1	Cytochrome P450 E1
